# 

**Published:** 1904-03-05

**Authors:** 


					TEE H0SPI1AL. "The Standard of highest purity *?Lancet. March 5th, 1904.
CADBURY'S M fc* CADBURY'S
COCOA % JU?> COCOA.
"A PERFECT FOOD." HO DRUGS OR ALKALIES USED.*
No. 910. Vol. XXXV. [S~] Satubday,
March 5th, 1904.
[ bUDscription lerms, j pR|CE THRE?PENCE|
L see p. 402.

				

